# Supplementary material for: A fault-intrusion-tolerant system and deadline-aware algorithm for scheduling scientific workflow in the cloud
Source: PeerJ Comput Sci. 2021 Nov 2;7:e747. doi: 10.7717/peerj-cs.747 (PMC8576554; doi:10.7717/peerj-cs.747)
Supplement: Supplemental Information 6 [file peerj-cs-07-747-s006.zip › Myproject1.1/docs/org/cloudbus/cloudsim/class-use/HarddriveStorage.html]

Uses of Class org.cloudbus.cloudsim.HarddriveStorage


JavaScript is disabled on your browser.


- Overview
- Package
- Class
- Use
- Tree
- Deprecated
- Index
- Help

- Prev
- Next

- Frames
- No Frames

- All Classes

## Uses of Class org.cloudbus.cloudsim.HarddriveStorage

- Packages that use HarddriveStorage

  | Package | Description |
  | --- | --- |
  |  |  |
  | --- | --- |
  | org.cloudbus.cloudsim |  |
  | org.workflowsim |  |
- - ### Uses of HarddriveStorage in org.cloudbus.cloudsim

    Subclasses of HarddriveStorage in org.cloudbus.cloudsim

    | Modifier and Type | Class and Description |
    | --- | --- |
    |  |  |
    | --- | --- |
    | `class` | `SanStorage` SANStorage represents a storage area network composed of a set of harddisks connected in a LAN. |
  - ### Uses of HarddriveStorage in org.workflowsim

    Subclasses of HarddriveStorage in org.workflowsim

    | Modifier and Type | Class and Description |
    | --- | --- |
    |  |  |
    | --- | --- |
    | `class` | `ClusterStorage` ClusterStorage is an extention of HarddriveStorage and it is used as a local storage system of a vm |

- Overview
- Package
- Class
- Use
- Tree
- Deprecated
- Index
- Help

- Prev
- Next

- Frames
- No Frames

- All Classes
